# Surgical Lymph Node Staging in Extremity Rhabdomyosarcoma: The E*p*SSG RMS 2005 Trial Experience

**DOI:** 10.1245/s10434-025-17908-3

**Published:** 2025-07-24

**Authors:** Sheila Terwisscha van Scheltinga, Johannes H. M. Merks, Florent Guerin, Timothy Rogers, Ross J. Craigie, Gabriela Guillén, Federica De Corti, Patrizia Dall’Igna, Raquel Dávila Fajardo, Gianni Bisogno, Andrea Ferrari, Daniel Orbach, Meriel Jenney, Julia C. Chisholm, Véronique Minard-Colin, Maya Cesen, Nina Jehanno, Laura S. Hiemcke-Jiwa, Ilaria Zanetti, Beatrice Coppadoro, Alida F. W. van der Steeg, Max M. van Noesel, Marc H. W. A. Wijnen

**Affiliations:** 1 Princess Máxima Center for Pediatric Oncology, Utrecht, The Netherlands; 2 Division of Imaging and Oncology, University Medical Center Utrecht, University of Utrecht, Utrecht, The Netherlands; 3 Department of Pediatric Surgery, Assistance Publique Hôpitaux de Paris, University Paris-Saclay, Le Kremlin Bicêtre, France; 4 University Hospitals Bristol and Weston NHS Foundation Trust, Bristol, UK; 5 Department of Pediatric Surgery, Royal Manchester Children’s Hospital, Manchester, UK; 6 Department of Pediatric Surgery, Pediatric Surgical Oncology Unit, Hospital Vall d’Hebron, Barcelona, Spain; 7 Pediatric Surgery, Women’s and Children’s Health Department, University Hospital of Padua, Padua, Italy; 8 Department of Radiation Oncology, University Medical Center Utrecht, Utrecht, The Netherlands; 9 Department of Women’s and Children’s Health, Pediatric Hematology Oncology Division, University Hospital of Padua, University of Padua, Padua, Italy; 10 Department of Pediatric Oncology, National Tumour Institute, Milan, Italy; 11 SIREDO Oncology Center (Care, Innovation and Research for Children, Adolescents and Young Adults with Cancer), Institut Curie, PSL University, Paris, France; 12 Department of Pediatric Oncology, University Hospital of Wales, Cardiff, UK; 13 Children and Young People’s Unit, Royal Marsden Hospital and Institute of Cancer Research, Sutton, UK; 14 Department of Pediatric and Adolescent Oncology, Gustave Roussy, Villejuif, France; 15 Pediatric Hematology and Oncology Department, University Children’s Hospital, Ljubliana, Slovenia; 16 Department of Nuclear Medicine, Institut Curie, Paris, France; 17 Department of Pathology, University Medical Center Utrecht, Utrecht, The Netherlands

**Keywords:** Rhabdomyosarcoma, Sentinel node, Staging, Lymph nodes, Biopsy, Extremity, Pediatric

## Abstract

**Background:**

The European pediatric soft tissue Sarcoma Study Group (E*p*SSG) RMS 2005 study recommends a lymph node biopsy for extremity rhabdomyosarcoma (RMS). The aim of our study was to analyze the results of the lymph node sampling strategies used, such as sentinel node biopsy (SNB) and nodal sampling (NS), and compare the outcome of patients undergoing different nodal staging techniques.

**Methods:**

All non-metastatic (M0) patients registered in the E*p*SSG RMS 2005 study with an RMS of the extremity, presenting between 2005 and 2016, were included for analysis of the lymph node sampling techniques used. The secondary objective was to compare the results and outcome for the different sampling procedures.

**Results:**

Of 198 patients, 144 had clinically/radiologically negative nodes (cN0), and 72/144 underwent a biopsy (26 SNB/46 NS). Final nodal status was upstaged to pN1 in 11/72 (15.3%) patients—6 after SNB and 5 after NS. In 54 radiologically malignant/suspicious-appearing nodes, 34 NS biopsies were performed, resulting in downstaging to N0 in 9/34 (26.5%) patients. 5-years overall survival (OS) of N0 patients versus N1 patients was 82.5% (95% confidence interval CI 74.7–88.0) versus 46.5% (95% CI 32.2–59.7). 5-years OS in N0 patients was not significantly different in biopsied and non-biopsied patients (*p* = 0.88). However, in N1 patients, survival was significantly better in biopsied compared with non-biopsied patients (*p* = 0.006).

**Conclusion:**

Lymph node staging plays a crucial role in determining appropriate treatment strategies. Pathology of sampled lymph nodes can upstage or downstage the lymph node status, guiding treatment decisions based on the stage.

**Supplementary Information:**

The online version contains supplementary material available at 10.1245/s10434-025-17908-3.

Lymph node metastases (N1) are an important adverse prognostic factor in extremity rhabdomyosarcoma (RMS).^1,2^ When involved, systemic treatment is intensified and radiotherapy (RT) of the involved lymph node(s) is indicated.^1–4^ Therefore, accurate staging of regional lymph nodes is mandatory for all extremity RMSs.^5^ When nodes are suspicious for disease on clinical or radiological examination, they should be biopsied. Due to the high rate (30–50%) of nodal involvement in extremity RMS, even when nodes are clinically negative, nodal biopsy remains mandatory.^5^

The Intergroup Rhabdomyosarcoma Study (IRS) IV showed that 17% of clinically negative nodes were found to harbor disease when biopsied. Furthermore, in the European pediatric soft tissue Sarcoma Study Group (E*p*SSG) RMS 2005 study on distal extremity RMS, a biopsy changed the nodal status in 20% of patients.^6,7^ Relapse has been reported to occur in 40% of extremity RMS, of which 63% are locoregional relapses, emphasizing the importance of accurate staging of the nodes;^1,2^ however, surgical compliance with systematic regional lymph node staging guidelines is poor.^8^

This study shows lymph node staging experience within the E*p*SSG RMS 2005 study. We evaluated the feasibility and results of the different techniques and their effect on outcome.

## Methods

All patients with non-metastatic extremity RMS enrolled in the E*p*SSG RMS 2005 study between October 2005 to December 2016 were included.^9^

### Protocol Guidelines for Staging and Treatment

Imaging of the primary site and regional lymph nodes consisted of magnetic resonance imaging (MRI), computed tomography (CT), and ultrasound; when available, 18F-fluorodeoxyglucose positron emission tomography (18F-FDG-PET)/CT was recommended. For extremity site (defined as extending from the shoulder girdle or buttock distally), systematic pathological analysis of regional lymph nodes was mandated in the protocol. Biopsy of clinically or radiologically suspicious lymph nodes (cN1) was recommended. In patients with clinically and radiologically normal lymph nodes (cN0), a sentinel node biopsy (SNB) was encouraged whenever feasible and available in treatment centers. However, in shoulder and buttock primary tumors, an SNB was often not performed because the regional nodes were not easy to reach surgically. If the SNB was not possible or available, random sampling or core needle biopsy of regional nodes was recommended.

A small subset of patients discussed in this paper were previously included in an earlier publication;^7^ this selection of patients had in-transit metastases, with the primary focus of that paper describing the clinical significance of in-transit nodes. In the present publication, we aimed to discuss the advantages and limitations of the various surgical lymph node staging techniques across regional lymph node basins.

In the E*p*SSG protocols, chemotherapy was applied according to risk stratification. In brief: VA (vincristine, D-actinomycin) for low-risk patients (embryonal histology [ERMS], IRS group I [radically resected], node-negative [N0], favorable size and age); and IVA (ifosfamide + VA) for the standard-risk group (ERMS, N0). High-risk patients (ERMS, >5 cm or >10 years, or alveolar histology [ARMS and N0]) were randomly assigned to IVA or IVADo (IVA + doxorubicin). When in complete remission after completion of IVADo, high-risk patients were eligible for a second randomization to either stop treatment or receive 24 weeks of maintenance chemotherapy (daily oral cyclophosphamide plus vinorelbine intravenously weekly for three out of each cycle of 4 weeks). Very high-risk patients (ARMS, N1) received the IVADo arm plus systematic maintenance therapy^10,11^ (electronic supplementary material [ESM] Table 1). Local treatment was achieved by surgery, RT, or both. The aim of local treatment was to cure the patient with no or minimal long-term sequelae.

**Table 1:** Clinical characteristics by lymph node exploration

	Sentinel node [*n* = 31]	Other biopsy [*n* = 80]	Total biopsy [*n* = 111]	No exploration [*n* = 87]	*N* = 198
*Age at diagnosis, years*					
< 10	21	62	83 (74.8)	66	149 (72.3)
≥ 10	10	18	28 (25.2)	21	49 (24.7)
Median age, years (range)	7.9 (0.3–18.5)	4.6 (0.1–20.8)		4.4 (0.01–23.9)	4.9 (0.01–23.9)
*Sex*					
Female	16	40	56 (50.5)	45	101 (51.0)
Male	15	40	55 (49.5)	42	97 (49.0)
*Histology*					
Alveolar RMS	25	55	80 (72.1)	52	132 (66.7)
Embryonal RMS	4	23	27 (24.3)	26	53 (26.8)
RMS NOS	–	1	1 (0.9)	2	3 (1.5)
Spindle cells/leiomyoma RMS	2	1	3 (2.7)	7	10 (5.1)
*Histology*					
Favorable RMS	6	24	30 (27.0)	33	63 (31.8)
Unfavorable RMS	25	56	81 (73.0)	54	135 (68.2)
*Fusion status*					
Negative	6	25	31 (27.9)	30	61 (30.8)
Positive	23	46	69 (62.1)	38	107 (54.0)
FOXO1 analysis not performed	2	9	11 (10.0)	19	30 (15.2)
*Lymph node involvement*					
N0	20	50	70 (63.1)	72	142 (71.7)
N1	11	30	41 (36.9)	15	56 (28.3)
*Extremity site*					
Distal	20	45	65 (58.6)	44	109 (55.1)
Proximal	11	35	46 (41.4)	43	89 (44.9)
*Extremity site*					
Lower	16	55	71 (64.0)	51	122 (61.6)
Upper	15	25	40 (36.0)	36	76 (38.4)
*Tumor primary site*					
Finger/hand/wrist	5	8	13 (11.7)	10	23 (11.6)
Forearm	8	10	18 (16.2)	14	32 (16.2)
Arm/elbow	2	7	9 (8.1)	4	13 (6.6)
Axilla/shoulder	–	–		8	8 (4.0)
Foot/ankle	–	10	10 (9.0)	10	20 (10.1)
Leg	7	17	24 ((21.6)	10	34 (17.2)
Thigh	9	19	28 (25.2)	20	48 (24.2)
Buttock/hip/inguinal	–	9	9 (7.2)	11	20 (10.1)
*Tumor size, cm*					
≤ 5	12	26	38	37	75 (37.9)
> 5	19	54	73	50	123 (62.1)
*T invasiveness*					
T1	24	66	90	63	153 (72.3)
T2	7	14	21	24	45 (22.7)
*IRS group*					
I	3	4	7	9	16 (8.1)
II	5	12	17	5	22 (11.1)
III	23	64	87	73	160 (80.8)
*Risk group*					
Low	2	–	2	2	4 (2.0)
Standard	2	7	9	12	21 (10.6)
High	16	45	61	59	120 (60.6)
Very high	11	28	39	14	53 (26.8)

Data are expressed as *n* or *n* (%) unless otherwise specified

*NOS* not otherwise specified, *RMS* rhabdomyosarcoma, *IRS* Intergroup Rhabdomyosarcoma Study

Primary resection of the tumor could be performed if clear resection margins could be obtained without loss of form and function.

Delayed surgery after a diagnostic biopsy was recommended after four courses of neoadjuvant chemotherapy. Postoperative RT (41.4 Gy and a boost if macroscopic incomplete resection) was delivered for tumors with alveolar histology and when the tumor extended to the resection margin. In some patients, preoperative RT was delivered, determined by the multidisciplinary team (MDT). Definitive RT (50.4 Gy) was delivered when conservative surgery was impossible.

Nodal disease received a dose of 41.4 Gy, with an RT boost when macroscopic tumor persisted after induction chemotherapy. In rare occasions when RT was contraindicated, a regional lymph node dissection could be considered.

### Definitions

Extremity site was defined as any part of the upper or lower extremity, buttock, or shoulder girdle. Regional lymph nodes are lymph nodes in the first draining nodal basin of the tumor site (ESM Table 2).

Lymph nodes involved clinically or radiologically were defined as cN1, and pN1 when confirmed with a biopsy, and unsuspicious clinical or radiological nodes were defined as cN0, and pN0 if pathology confirmed the absence of nodal involvement. When nodal sampling (NS) was performed for imaging-negative patients, this was defined as random NS (rNS), or if an SNB was performed, this was defined as SNB. When sampling was performed for imaging-positive patients, this was described as targeted NS (tNS)

Any nodal spread between the primary tumor and regional lymph nodes, or between two regional lymph node basins, was defined as ‘in-transit’ N1 spread. All pathological lymph nodes beyond regional lymph nodes were considered distant metastases (M1).

The sentinel node (first-tier node, first-echelon node) is the first lymph node on the direct drainage pathway from the primary tumor. From a surgeon’s perspective, a sentinel node was defined as a lymph node containing blue dye and/or a radiotracer. A non-sentinel node was a lymph node, without blue dye or radiotracer uptake, biopsied during the same procedure.^12^ A positive sentinel node was defined as a sentinel lymph node containing tumor cells on pathological examination (pN1). In the E*p*SSG study, the sentinel node procedures were indicated for cN0 patients. For this study, we included only the SNB performed in clinically/imaging-negative patients for analysis of the performance of this technique.

### Statistics

Primary endpoints were the feasibility of the lymph node sampling procedures and their effect on definitive staging. Calculation of sensitivity and specificity of the different procedures was not possible because of the absence of a golden standard (resection of all lymph nodes), as performed during the introduction of the SNB in breast cancer and melanoma.^13–15^

Secondary endpoints for analysis were 5-years event-free survival (EFS) and 5-years overall survival (OS). EFS was defined as the time from diagnosis to first event (relapse, disease progression, secondary malignancy, or death from any cause), while OS was defined as the time from diagnosis to latest follow-up or death from any cause. The survival curves were estimated using Kaplan–Meier analysis. Statistical calculations were performed using SAS software (release 9.4; SAS Institute Inc., Cary, NC, USA).

## Results

Between October 2005 and December 2016, 198 patients with localized non-metastatic RMS of the extremity were registered. Patients had a median age of 4.9 years (range 2–23.9), and median follow-up for alive patients (*n* = 140) was 73.6 months from diagnosis (range 14.0–158.9). Patient and tumor characteristics are listed in Table 1.

### Lymph Node Staging

**Fig. 1:**
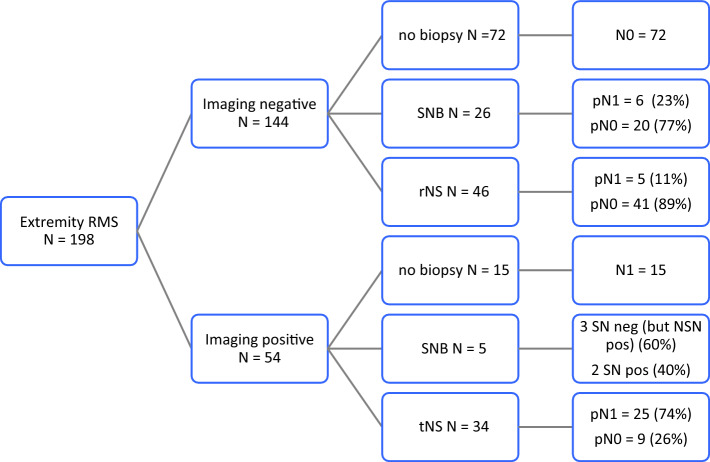
Results of biopsy in clinically/radiologically negative and positive patients. *SNB* sentinel node biopsy, *rNS* random nodal sampling, *tNS* targeted nodal sampling, *SN* sentinel node, *NSN* non-sentinel node, *RMS* rhabdomyosarcoma, *neg* negative, *pos* positive

Malignant nodal status (cN1 and/or pN1) was assigned prior to the initiation of chemotherapy in 56/198 (28.3%) patients. In 87/198 patients, nodal status was concluded based solely on imaging. A nodal biopsy was performed at diagnosis in 111/198 patients, including NS in 74 patients, an SNB in 31 patients, and a needle biopsy in 6 patients. Of the 198 patients, 144 had radiology findings not suspicious for disease (cN0); half of these 144 (*n* = 72) patients underwent a biopsy. rNS was performed in 46 patients, with pathology confirming pN0 status in 41 patients, and staging 5 patients as pN1. In 26 cN0 patients who underwent a sentinel node procedure, pN0 status was confirmed in 20 patients, while 6 patients were staged as pN1 (Fig. 1).

Of 54 patients with suspected nodal involvement on radiology (cN1), biopsies were performed in 39 (72%) patients. Among these, tNS was performed in 34 patients, confirming pN1 status in 25 patients; in the remaining 9 patients, the lymph nodes were found to be tumor-negative (pN0). For the remaining 5/39 patients, a sentinel node procedure was erroneously performed. This confirmed N1 status in 2 patients, while the remaining 3/5 patients had a false negative result. Targeted biopsies from other sites were positive in these 3 cases.

In 15/54 patients with clinically/radiologically positive nodes, biopsies were not performed, for the following reasons: a large lymph node mass in 3 patients (obvious involvement), a difficult sampling area in 2 patients (around the internal and external iliac vessels and caval vein), and not specified in 1 patient. For the remaining 9 patients staged as N1, the lymph nodes were smaller than 15 mm and should have been biopsied according to protocol. Figure 1 shows the results of the nodal biopsies in both imaging-negative and imaging-positive patients.

### Sentinel Node Biopsy

SNB was performed in 31 patients, confined to six treating centers. The SNB was indicated in 26 patients, and was incorrectly performed in 5 patients because positive nodes were present at imaging. Radiocolloid was used in 29/31 patients, with the addition of blue dye in 12/29 patients. In 1/31 patients, blue dye was used as a single tracer, and the SNB technique was unknown in 1 patient. A median of two sentinel nodes were harvested (range 1–5). The sentinel node upstaged 6/26 (26%) patients with imaging-negative nodes.

### Nodal Sampling

In 34/80 patients with node sampling, nodes were suspicious or clearly involved on imaging (cN1). A targeted biopsy downstaged 9/34 (26.5%) patients from suspicious cN1 to pN0, and confirmed the N1 status in 25. Patients were upstaged in 5/46 radiologically negative nodes by random node picking, from cN0 to pN1 (10.9%).

Regional nodal sites per primary site are listed in ESM Table 2.

**Table 2:** Events in cN0 and pN0 patients, categorized by lymph node exploration technique

	Sentinel node [*n* = 20]	Nodal sampling [*n* = 50]	No exploration [*n* = 72]	Total [*N* = 142]	*p*-value
No event	14 (70.0)	27 (54.0)	49 (68.1)	90 (63.4)	0.2286
Event	6 (30.0)	23 (46.0)	23 (31.9)	52 (36.6)	
Local relapse (LR)	–	5	6	11	
Regional lymph node (N)	–	3	4	7	
LR + N	–	2	2	4	
Metastases relapse (MTS)	3	10	3	16	
LR + MTS	–	–	1	1	
LR + N + MTS	1	–	–	1	
N + MTS	1	–	1	2	
Progressive disease	1	2	3	6	
Second tumor	–	1	3	4	

Data are expressed as *n* or *n (%)*

### Treatment

All patients received risk-stratified chemotherapy. Delayed surgery on the nodes was performed in 24 patients (sampling in 15, dissection in 7; 2 patients had unknown procedures). Lymph node areas were treated with RT in 47/56 N1 patients. Reasons for omitting RT on the nodes included young age and amputation of the limb, including the nodes area.

### Outcome

Tables 2 and 3 show the events occurring in patients, according to the different staging techniques. An event occurred in 84/198 (42.4%) patients, and 58/84 (69%) patients died. pN0 biopsied patients relapsed in 29/70 (41.4%) cases. A relapse confined to the nodes occurred in 3/50 biopsied patients and 4/72 non-biopsied (cN0) patients. In the node-positive (N1) patients, 32/56 (57.1%) had a relapse. A relapse confined to the nodes occurred in 7/41 biopsied patients and 1/15 non-biopsied patients. Eleven of 49 N1 patients treated with locoregional RT relapsed. Four patients in whom nodal basins were treated with RT relapsed within the RT field, and 6 patients relapsed outside of the RT field. In 1 patient with relapse, insufficient data were available to determine the relationship with receipt of RT. Only 4/32 (12.5%) pN1 patients who developed an event, survived.

**Table 3:** Events in cN1 and pN1 patients, categorized by lymph node exploration technique

	Sentinel node [*n *= 6]	Other biopsy [*n *= 35]	No exploration [*n *= 15]	*N* = 56	*p*-value
No event	3 (50.0)	16 (45.7)	5 (33.3)	24 (42.9)	0.7148
Event	3 (50.0)	19 (54.3)	10 (66.7)	32 (57.1)	
Local relapse (LR)	–	4	1	5	
Regional lymph node (N)	1	6	1	8	
Metastases relapse (MTS)	2	4	6	12	
LR + MTS	–	1	–	1	
LR + N + MTS	–	1	–	1	
N + MTS	–	2	–	2	
Progressive disease	–	1	2	3	

**Fig. 2:**
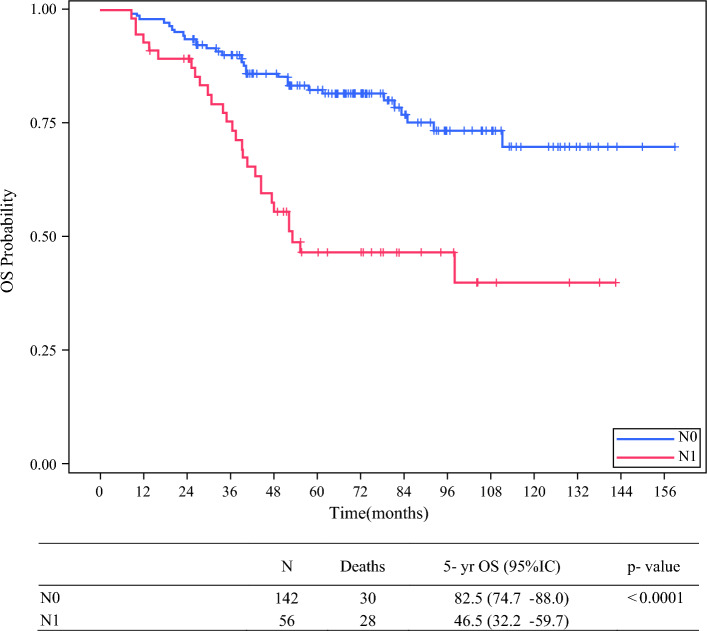
Overall survival by lymph node stage, *5-yr* 5-years, *OS* overall survival, *CI* confidence interval

**Fig. 3:**
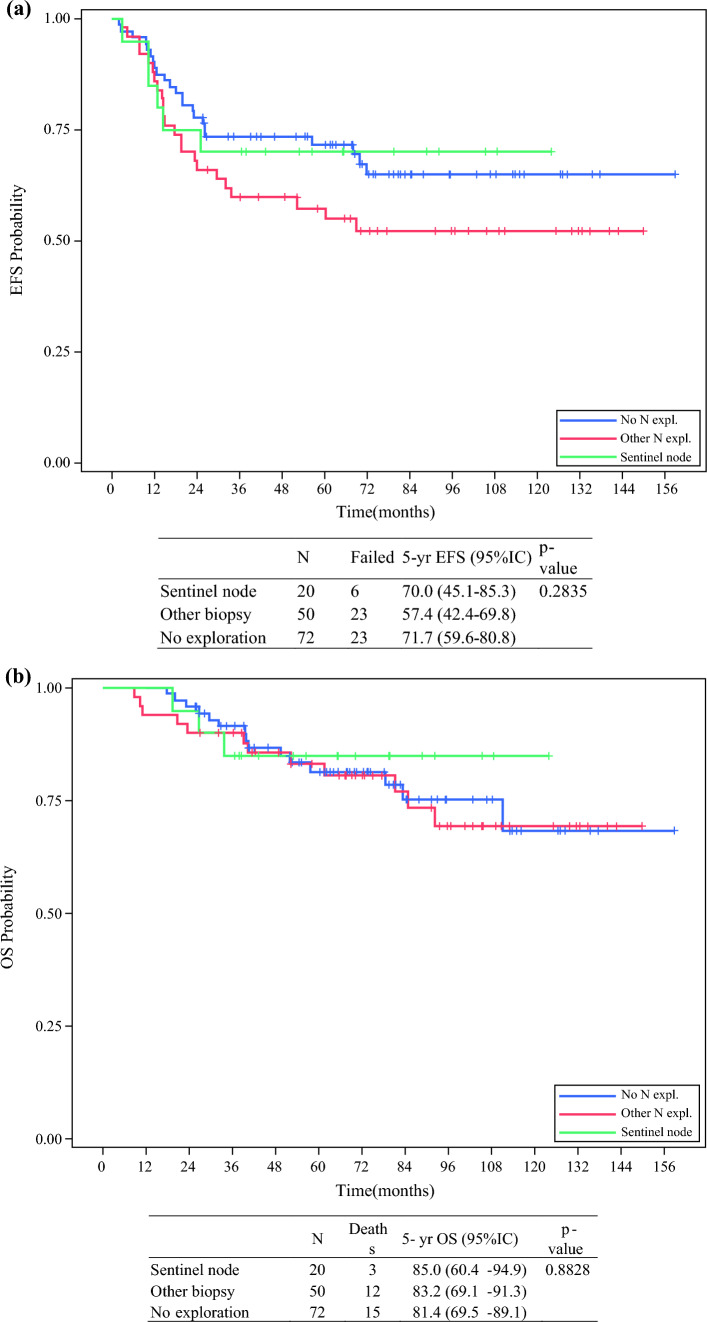
**a** Event-free survival by lymph node exploration in N0 and pN0. **b** Overall survival by lymph node exploration in N0 and pN0 patients, *5-yr* 5-years, *OS* overall survival, *CI* confidence interval

**Fig. 4:**
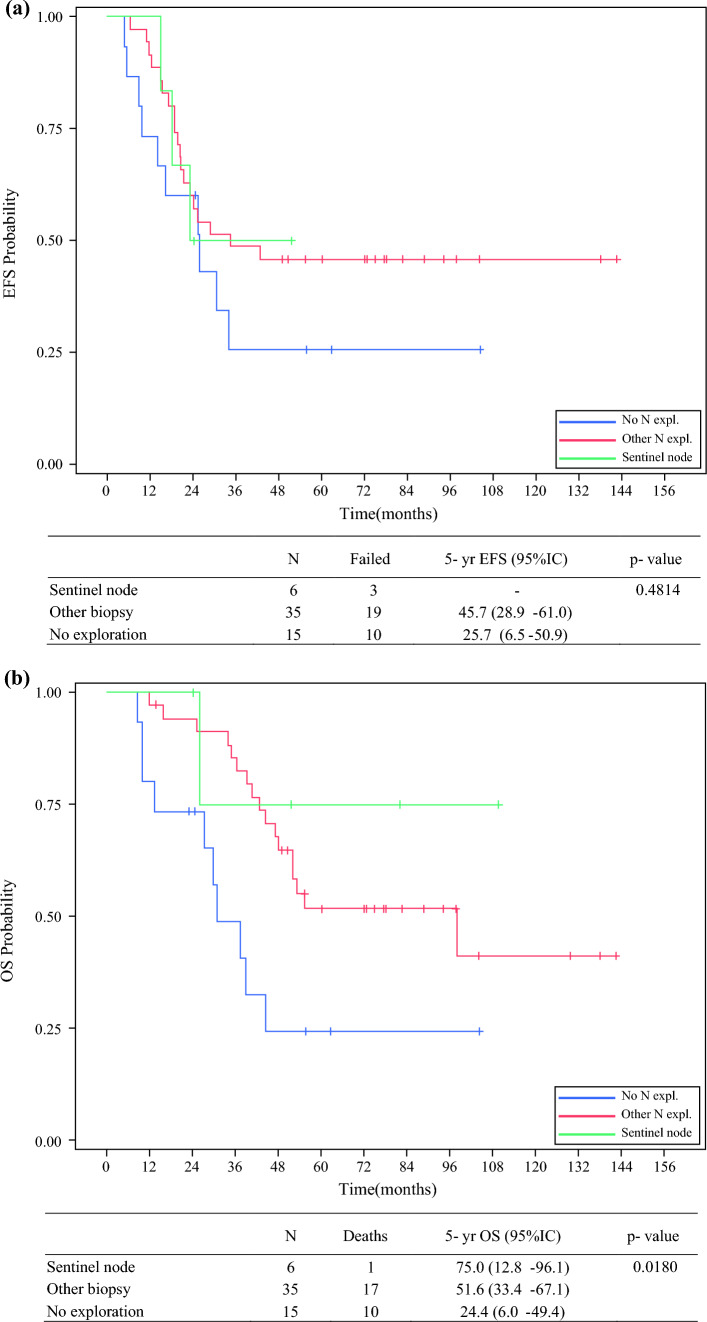
**a** Event-free survival by lymph node exploration in N1 and pN1 patients. **b** Overall survival by lymph node exploration in N1 and pN1 patients, *5-yr* 5-years, *OS* overall survival, *CI* confidence interval

EFS and OS were significantly worse in N1 versus N0 patients (Fig. 2). Figures 3 and 4 show the EFS and OS in N0 and N1 patients, by type of biopsy. OS in N1 patients staged with any type of biopsy (*n* = 41) was significantly better than in N1 patients only staged with imaging (*n* = 15): 53.8% (95% CI 36.4–68.4) versus 24.4% (95% CI 6.0–49.4) [*p* = 0.006].

## Discussion

In this large, consecutive cohort, we assessed how regional nodal staging procedures influenced the staging of patients with extremity RMS. Although a biopsy of the nodes was strongly recommended for patients included in the E*p*SSG RMS 2005 protocol, almost half of the patients with radiological-negative nodes and one-third of patients with radiological-positive nodes were not biopsied. For cN0 patients, the reluctance to perform random NS might be explained by the estimated a priori high chance of a false negative result; however, NS changed nodal status from cN0 to pN1 in 15.3% of cases. In addition, we demonstrated that the sentinel node procedure is a feasible, structured, and accurate alternative for random node sampling for patients with clinical and imaging-negative nodes. However, an SNB was only performed in 26/144 cN0 patients, illustrating the limited availability of the technique across pediatric oncology centers.

Although the SNB is contraindicated in patients with radiologically positive nodes, a SNB was still performed in 5 cN1 patients. This led to a false negative result in 3/5 patients, due to the fact that an involved lymph node can alter lymph drainage, leading to a false negative sentinel node.^14^ These patients should receive a targeted nodal biopsy.

The sentinel node procedure used in patients with radiology-negative nodes can identify small lymph node metastases, with only minor infiltration of malignant cells (micrometastases), only detectable through pathology. This might be a different category of pN1 as it is currently unclear how micrometastases in regional lymph nodes influence survival. However, in paratesticular patients >10 years of age, more than 30% of N0 patients staged with imaging alone had a relapse in the nodes, while none of the N1 patients, treated with RT on the nodes, relapsed.^16^. This suggests that microscopic nodal metastases do impact survival and may be undertreated by not applying appropriate local therapy. In our study, only 6 patients staged with a sentinel node procedure were pN1, making it difficult to draw definitive conclusions.

An important finding is that 26.5% of imaging-suspicious nodes were actually node-negative when sampled. Local treatment of the nodes and intensified systemic therapy can be omitted in most patients because they are then allocated to a lower-risk group treatment regimen; therefore, targeted biopsy of suspicious nodes is essential.

On the contrary, a lymph node biopsy in cN0 patients can also lead to upstaging, resulting in intensified systemic and local treatment in 15.3% of our patients.

Although regional lymph node involvement (N1) in extremity RMS is widely recognized as an adverse prognostic factor, the evidence supporting treatment intensification in this setting is primarily derived from retrospective analyses and subgroup observations within prospective studies. Intensified systemic therapy and locoregional RT are protocol-based recommendations in the EpSSG RMS 2005 study and other cooperative group protocols.^1–4^ However, to date, no prospective randomized trials have specifically evaluated treatment intensification in node-positive patients. The International Society of Pediatric Oncology (SIOP) Malignant Mesenchymal Tumor (MMT) Group 89 study (SIOP-MMT89) explored increased dose-intensity of ifosfamide (no benefit) and intensified (six-drug) chemotherapy in regional node disease. The results for this group of patients in MMT89 showed improved survival compared with MMT84 (5-years OS: 60% vs. 42%; EFS: 51% vs. 42%).^17^

In our cohort, treatment was intensified in most patients with nodal involvement, yet EFS and OS remained inferior compared with node-negative patients. Notably, we observed significantly improved survival among N1 patients who underwent nodal biopsy, as opposed to those staged solely by imaging. Several factors may contribute to this finding. In patients with large, radiologically evident nodes, biopsy was often omitted due to presumed disease involvement; these patients may have had a higher tumor burden, contributing to poorer outcomes. This is supported by the more favorable outcomes observed in patients staged with SNB, in whom micrometastatic disease was identified (Fig. 4b). An alternative explanation is that biopsy may contribute to improved locoregional control by reducing nodal tumor burden. While prospective evidence for survival improvement through treatment intensification in N1 patients is still lacking, current clinical protocols continue to support this approach, based on the consistent association between nodal involvement and poor prognosis, and on indirect evidence suggesting improved outcomes when appropriate locoregional therapies are applied.

Our study comes with multiple limitations. Although it is the largest and most recent cohort of consecutively treated patients within the concept of a prospective clinical trial to date, surgical lymph node staging guidelines were frequently not adhered to. Inherently, numbers for the different sampling techniques used were relatively small, only allowing for more explorative analysis. Thus far, the SNB technique is mainly used in extremity RMS; however, head and neck, perineal, and paratesticular RMS also have a high propensity to metastasize to the lymph nodes. The sentinel node procedure could also be of value in these sites,^18,19^ but a critical view on the technique, as well as tracer options, are to be discussed. The use of [^68^Ga]-tilmanocept PET/CT lymphoscintigraphy or a peroperative fluorescent tracer show promising results but are not yet established techniques.^20–23^ International collaboration with adequate registration of techniques used is important to implement and evaluate new techniques and applications.

## Conclusion

Lymph node metastases are an important prognostic factor in extremity RMS. To ensure proper nodal staging, a biopsy is essential. For detecting involved lymph nodes, the optimal approach involves targeted NS techniques for radiological suspicious nodes; for radiologically unsuspicious nodes, the sentinel node technique is a structured and feasible technique, and for centers where sentinel node procedures are not available, random NS is highly recommended.

## Supplementary Information

Below is the link to the electronic supplementary material.

Supplementary file 1 (DOCX 16 KB)
