# Supplementary material for: Surgical Lymph Node Staging in Extremity Rhabdomyosarcoma: The EpSSG RMS 2005 Trial Experience
Source: Ann Surg Oncol. 2025 Jul 24;32(10):7751–61. doi: 10.1245/s10434-025-17908-3 (PMC12454586; doi:10.1245/s10434-025-17908-3)
Supplement: Supplementary file 1 — (DOCX 16 KB) [file 10434_2025_17908_MOESM1_ESM.docx]

**SUPPLEMENTARY TABLE 1** Detailed chemotherapy regimens.

| Chemotherapy regimen | *N* = 198 |
| --- | --- |
| IVA | 60 |
| IVA in combination with VA or VAC | 15 |
| IVADo + IVA/VA | 33 |
| IVADo + IVA + maintenance | 59 |
| IVA + maintenance | 26 |
| Other | 5 |

*I* ifosfamide, *V* vincristine, *A* actinomycin, *Do* doxorubicin, *VNL/CPM* vinorelbine/ cyclophosphamide (maintenance therapy)

**SUPPLEMENTARY TABLE 2** Regional nodes per primary tumor site

| Primary tumor site | Regional lymph node basin |
| --- | --- |
| Hand and arm | Epitrochlear  Axilla |
| Upper arm | Axilla |
| Shoulder | Axilla  Subclavian |
| Foot and lower leg | Popliteal  Inguinal |
| Thigh | Inguinal  Iliacal (adductor part) |
| Buttock | Inguinal  Iliacal |

**SUPPLEMENTARY TABLE 3** Biopsied nodal sites and pathological result per primary tumor site

| Primary site | Biopsied Ln site | SNB  *N* (pN1) | Node sampling  *N* (pN1) | Needle biopsy  *N* (pN1) | Total biopsies  *N* (pN1) |
| --- | --- | --- | --- | --- | --- |
| Foot/ankle | Inguinal  popliteal  Inguinal+popliteal  Inguinal+iliacal  Unknown | 0  0  0  0  0 | 4 (1)  1 (1)  1 (0)  1 (1)  2 (0) | 1 (1) | 5 (2)  1 (1)  1 (0)  1 (1)  2 (0) |
| Leg | Inguinal  Popliteal  In-transit  Inguinal+popliteal | 3 (1)  3 (1)  0  1 (1) | 8 (1)  4 (1)  1 (1)  3 (3) | 1 (1) | 11 (2)  7 (2)  1 (1)  5 (5) |
| Thigh | Inguinal  Inguinal + iliacal  In transit  Unknown | 9 (1) | 13 (1)  1 (0)  1 (0)  3 (0) | 1 (0) | 23 (2)  1 (0)  1 (0)  3 (0) |
| Buttock/groin | Inguinal  Iliacal  Inguinal+Iliacal  Unknown | 0  0  0  0 | 4 (2)  1 (1)  1 (0)  2 (0) | 1 (1) | 4 (2)  1 (1)  2 (1)  2 (0) |
|  |  |  |  |  |  |
| Hand/wrist | Axilla  Epitrochlear  Axilla+epitrochlear  Unknown | 5 (2) | 5 (3)  0  1 (1)  1 (0) | 1 (1) | 11 (6)  0  1 (1)  1 (0) |
| Forearm | Axilla  Epitrochlear  Axilla+epitrochlear  Unknown | 7 (2)  0  1 (0)  0 | 7 (3)  1 (1)  0  2 (0) |  | 14 (5)  1 (1)  1 (0)  2 (0) |
| Upper arm/elbow | Axilla  In transit  Axilla+in-transit  Unknown | 2 (1) | 4 (1)  1 (1)  0  1 (0) | 1 (1) | 6 (2)  1 (1)  1 (1)  1 (0) |
| Shoulder/axilla |  | 0 | 0 |  | 0 |
| Total |  | 31 (9) | 74 (23) | 6 (5) | 111 (37) |
